# A novel way of managing shearing of epidural catheter during tunnelling

**DOI:** 10.4103/0019-5049.72663

**Published:** 2010

**Authors:** Kamal Kishore, Sandeep Sahu, Manish Kumar Singh, Anil Agarwal, PK Singh

**Affiliations:** Department of Anaesthesiology, Sanjay Gandhi Post Graduate Institute of Medical Sciences, Lucknow, Uttar Pradesh, India

Sir

Subcutaneous tunnelling of the epidural catheter is routinely practiced for anchoring the epidural catheter. There are different techniques for subcutaneous tunnelling which are associated with complications like needle stick to the clinician or shearing of the epidural catheter. Rose GL (2009) reported the efficacy of needle sheath for prevention of these complications.[1]

**Figure 1:**
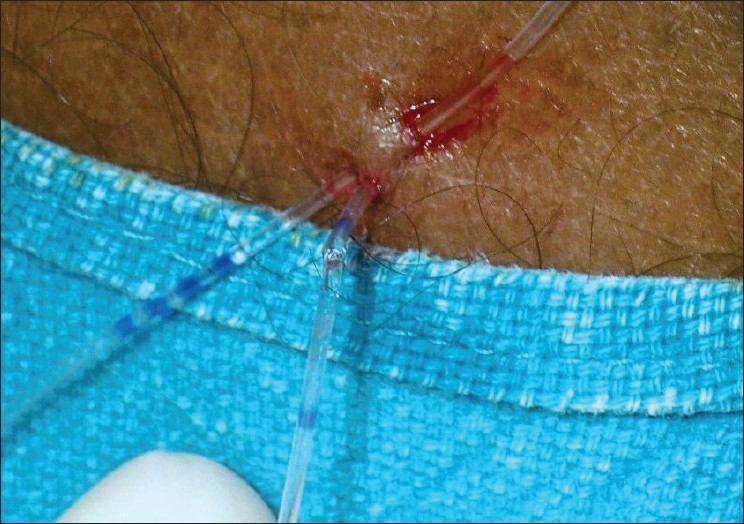
Shearing of epidural catheter during subcutaneous tunnelling

**Figure 2:**
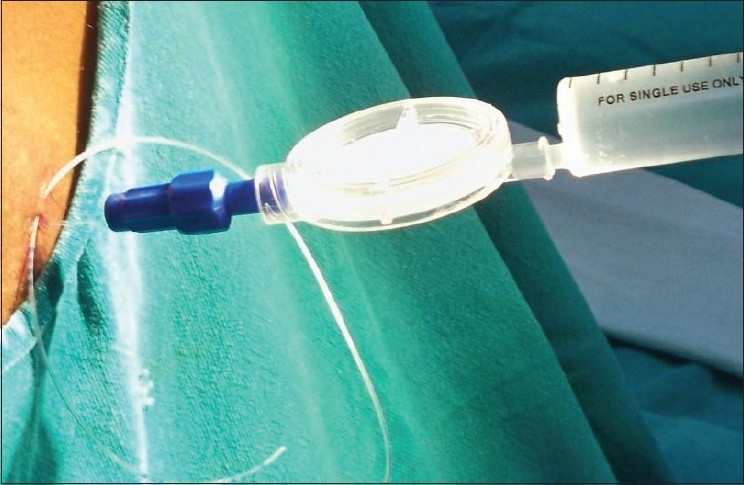
Cut end of the catheter at the point of shearing, which was attached to the catheter connector and filter assembly

Still, the likelihood of shearing of the epidural catheter cannot be ruled out. Following shearing of the epidural catheter it has to be pulled out, as it cannot be used for administering medication.[2] Recently, we came across shearing of epidural catheter during subcutaneous tunnelling [Figure 1]. We did not pull out the epidural catheter; rather we cut the catheter at the point of shearing and attached the cut end of the catheter (near the point of exit from the back of the patient) to the catheter connector and filter assembly. Thereafter we administered medication through this assembly that was later fixed to the back of the patient [Figure 2]. Subsequently a highpressure, low-volume extension tube was attached to the assembly, which was used for administration of drugs for the management of postoperative pain. We could safely and effectively manage the patient intra-operatively and took care of his postoperative pain (three days) without the need of replacing the epidural catheter thereby saving time and money.

Fortunately, shearing of the catheter happened at an extracutaneous site so we got extra length to attach the filter and other assembly with it. Although, short length of catheter or slightly bulky assembly (filter system) or absence of loop may pose a possibility of dislodgement it is a safe and cost-effective method. We therefore suggest that this technique could be employed in cases of shearing of epidural catheter during the process of subcutaneous tunnelling.
